# Inhibitory Effect of Apomorphine on Focal and Nonfocal Plasticity in the Human Motor Cortex

**DOI:** 10.3390/pharmaceutics13050718

**Published:** 2021-05-13

**Authors:** Shane M. Fresnoza, Giorgi Batsikadze, Lynn Elena Müller, Constanze Rost, Michael Chamoun, Walter Paulus, Min-Fang Kuo, Michael A. Nitsche

**Affiliations:** 1 Institute of Psychology, University of Graz, 8010 Graz, Austria; 2 BioTechMed, 8010 Graz, Austria; 3 Department of Clinical Neurophysiology, Georg-August-University, 37075 Göttingen, Germany; lynn.mueller@gmx.de (L.E.M.); C.Rost@vincenz.de (C.R.); malke83@gmx.de (M.C.); wpaulus@med.uni-goettingen.de (W.P.); nitsche@ifado.de (M.A.N.); 4 Department of Neurology, University of Duisburg-Essen, 45147 Essen, Germany; giorgi.batsikadze@uk-essen.de; 5 Department of Psychology and Neurosciences, Leibniz Research Centre for Working Environment and Human Factors, 44139 Dortmund, Germany; Kuo@ifado.de; 6 Department of Neurology, Bergmannsheil, Ruhr-University-Bochum, 44789 Bochum, Germany

**Keywords:** dopamine, plasticity, motor cortex, apomorphine: transcranial direct current stimulation, paired associative stimulation, motor evoked potential, transcranial magnetic stimulation

## Abstract

Dopamine is crucial for neuroplasticity, which is considered to be the neurophysiological foundation of learning and memory. The specific effect of dopamine on plasticity such as long-term potentiation (LTP) and long-term depression (LTD) is determined by receptor subtype specificity, concentration level, and the kind of plasticity induction technique. In healthy human subjects, the dopamine precursor levodopa (L-DOPA) exerts a dosage-dependent non-linear effect on motor cortex plasticity. Low and high dosage L-DOPA impaired or abolished plasticity, while medium-dose preserved and reversed plasticity in previous studies. Similar dosage-dependent effects were also observed for selective D1-like and D2-like receptor activation that favor excitatory and inhibitory plasticity, respectively. However, such a dosage-dependent effect has not been explored for a nonselective dopamine agonist such as apomorphine in humans. To this aim, nonfocal and focal motor cortex plasticity induction using paired associative stimulation (PAS) and transcranial direct current stimulation (tDCS) were performed respectively in healthy participants under 0.1, 0.2, 0.3 mg apomorphine or placebo drug. Transcranial magnetic stimulation-elicited motor-evoked potentials were used to monitor motor cortical excitability alterations. We hypothesized that, similar to L-DOPA, apomorphine will affect motor cortex plasticity. The results showed that apomorphine with the applied dosages has an inhibitory effect for focal and nonfocal LTP-like and LTD-like plasticity, which was either abolished, diminished or reversed. The detrimental effect on plasticity induction under all dosages of apomorphine suggests a predominantly presynaptic mechanism of action of these dosages.

## 1. Introduction

Dopamine is crucial for the voluntary control of movement, learning, and memory processes. The neurophysiological basis of its effect on learning (including motor learning) and memory, as shown in animal in vitro and in vivo experiments, is its modulating effect on the formation and maintenance of plasticity, namely long-term potentiation (LTP) and long-term depression (LTD) [1,2]. In animals, stimulation of the ventral tegmental area (VTA) at a frequency known to evoke dopamine enhancement in the prefrontal cortex produces a long-lasting enhancement of the magnitude of hippocampal-prefrontal cortex LTP, whereas cortical dopamine depletion generates a decrease of LTP [3].

In the human primary motor cortex (M1), induction of LTP- and LTD-like plasticity that share physiological mechanisms, including calcium-, and N-methyl-D-aspartate (NMDA) receptor-dependency with plasticity induced in animal models is feasible by non-invasive brain stimulation (NIBS) techniques such as transcranial direct current stimulation (tDCS), paired associative stimulation (PAS) and repetitive transcranial magnetic stimulation (rTMS) [4,5,6,7]. In PAS, somatosensory-motor cortex plasticity is induced by pairing transcranial magnetic stimulation (TMS) of M1 with electrical stimulation of a peripheral mixed nerve [4]. Based on the concept of spike-time dependent plasticity, an inter-stimulus-interval of 25 ms results in synchronous arrival of both inputs at the level of M1 and induction of LTP-like plasticity, while an inter-stimulus-interval of 10 ms causes asynchronous stimulation of M1 and induction of LTD-like plasticity. On the other hand, tDCS involves the application of low-intensity (1–2 mA) electrical current through the intact head to modulate excitability and activity of cortical neurons [8]. During stimulation, tDCS modulates neuronal excitability by altering neuronal membrane potentials caused by the opening or closing of voltage-gated ion channels [9]. The effect of tDCS is polarity-dependent, anodal (positive current) and cathodal (negative current) stimulation results in subthreshold neuronal membrane depolarization (which increases the likelihood of neuronal firing) or hyperpolarization (which decreases the likelihood of neuronal firing), respectively. With stimulation for some minutes, the effects of stimulation persist after intervention and are considered to represent LTP-like (for anodal) and LTD-like (for cathodal) plasticity [10]. Both plasticity-inducing stimulation protocols induce long-lasting, NMDA receptor-dependent neuroplastic excitability changes. However, the main difference lies in more focal effects on a restricted subgroup of synapses induced by PAS, as opposed to the plasticity induced by tDCS, which is synaptically driven but not limited to specific subgroups of synapses. PAS-induced plasticity is also associative and timing-dependent, compared with the tonic neuronal polarization induced by tDCS [7,11,12].

Plasticity induction in M1 also depends on intact dopamine signaling because tDCS and PAS, as well as theta-burst stimulation (TBS)-induced neuroplastic neuronal excitability changes (as monitored by TMS-evoked MEPs) are impaired by dopamine receptor antagonists in healthy humans [13,14,15]. Moreover, alterations of dopaminergic pathways in neuropsychiatric conditions such as Parkinson’s disease (PD) and schizophrenia also compromise plasticity [16,17,18]. However, to date, the complex neurophysiological mechanisms underlying the impact of dopamine on neuroplasticity and cognitive functions are not yet fully understood. As observed in animal and human studies, the impact of dopamine depends on several factors such as receptor subtype, concentration level/dosage of dopamine, and type of plasticity induction [12,19]. In NIBS studies involving healthy humans, the dopamine precursor levodopa (L-DOPA) exerts an inverted-U-shaped effect on nonfocal and focal plasticity induced by tDCS and PAS in M1, respectively. Low and high dose L-DOPA impaired or abolished the induction of LTP- and LTD-like plasticity. In contrast, for medium dose L-DOPA, nonfocal LTD-like plasticity and focal LTP- and LTD-like plasticity are facilitated, while nonfocal LTP-like plasticity is converted to LTD [20,21]. A similar pattern of results was obtained by the selective D2 receptor agonist bromocriptine [22]. On the other hand, enhancing D1-like receptor activity by combining L-DOPA and the D2 receptor antagonist sulpiride facilitated tDCS- and PAS-generated LTP-like effects at medium, but not low and high dosages [23], whereas it abolished or trendwise converted LTD-like plasticity into facilitation, largely independent from dosage, and plasticity induction protocol [23]. Thus, it seems that the effects of L-DOPA on M1 plasticity are primarily transmitted via the D2 receptor.

Direct comparisons of the effects of different dopaminergic agents on plasticity are, however, not trivial because they affect dopamine receptors in different ways not only with respect to receptor subtype specificity. L-DOPA is a dopamine precursor taken up by dopaminergic neurons, converted to dopamine, and released into the synaptic cleft dependent on the activity of the respective neurons [24,25]. In contrast, dopamine receptor agonists, such as bromocriptine, a D2 receptor agonist, bind directly to postsynaptic receptors independent of neuronal activity. It is thus relevant to compare the effect of L-DOPA with broad-spectrum dopamine receptor agonists, such as apomorphine, which binds at the same receptors as dopamine with similar affinity [26], but in a neuronal activity-independent way. Physiologically and behaviorally, previous studies reported grossly comparable effects of both substances. In small doses, they decrease the firing rate of dopaminergic neurons in rats [27]. Their effects on hand tapping scores, walking time, tremor severity, dyskinesia scores, and scores of the modified Webster disability scale did not differ in PD patients [26,28]. However, also in PD patients, discernable effects on more complex cognitive processes such as visuospatial working memory, where L-DOPA and apomorphine had an enhancing and detrimental effect, respectively, were observed [29]. Thus, by now, it cannot be excluded that different effects of L-DOPA and dopamine receptor subtype agonists on plasticity are not completely caused by receptor subtype specificities of these agents but also pharmacokinetic aspects.

In the present study, we thus aimed to explore the effect of the unspecific dopamine agonist apomorphine on M1 plasticity in healthy humans. In two separate experiments, we tested the impact of apomorphine on nonfocal plasticity induced by tDCS (Experiment 1) and focal plasticity induced by PAS (Experiment 2). Based on available studies, we hypothesized that apomorphine would exert an induction protocol-dependent effect on M1 plasticity similar to L-DOPA.

## 2. Materials and Methods

### 2.1. Participants

For Experiment 1, 24 healthy young adults were recruited and randomly assigned to a group that received anodal (8 males, 4 females; mean age ± SD: 26.50 ± 3.15 years), or cathodal tDCS (5 males, 7 females; mean age ± SD: 26.83 ± 4.99 years). For Experiment 2, 12 additional healthy young adults were recruited (6 males, 6 females, mean age ± SD: 28.33 ± 4.46 years) and each received PAS10, and PAS25. A priori power calculations indicated that in Experiment 1 with a repeated measure (with between-within interactions) design, a sample size of 24 is sufficient to achieve a statistical power (1-β) of 90% at an alpha level of 0.05 and a moderate effect size (0.50) (G*Power 3.1.9.2) for the primary statistical test, a mixed model ANOVA. For Experiment 2 with a repeated measure (within-subject) design, a sample size of 12 is sufficient to achieve the same statistical power for the primary statistical test, a repeated measure ANOVA. All participants were right-handed, according to the Edinburgh Handedness Inventory [30]. Exclusion criteria included a history of acute or chronic medical or neuropsychiatric disorders (e.g., depression, epilepsy, and stroke), neurosurgical interventions, intake of medications two weeks before the study, substance abuse, contraindications to tDCS such as metallic or electrical implants in the body or the head, and age below 18 or above 40 years. A pregnancy test ruled out pregnancy. Occasional smokers were allowed to participate, but all participants were instructed to avoid alcoholic drinks at least 24 h before the experiments. The Ethics Committee of the University of Göttingen approved the study, and all procedures conform to the Declaration of Helsinki regarding human experimentation. Written informed consent was obtained from each participant.

### 2.2. Monitoring of Corticospinal Excitability

The impact of apomorphine on corticospinal excitability was monitored via motor-evoked potentials (MEPs) generated by TMS over the M1 representation of the right abductor digiti minimi muscle (ADM). The optimal coil position (site at which stimulation resulted in the largest MEP amplitudes with a given moderate TMS intensity) was first determined using single-pulse TMS generated by a Magstim 200 magnetic stimulator (The Magstim Co. Ltd., Whitland, UK) at a frequency of 0.25 Hz via a figure-of-eight magnetic coil (diameter of one winding: 70 mm, peak magnetic field: 2.2 Tesla). To generate a posterior–anterior current direction in the brain aligned to the orientation of the precentral gyrus, the coil was held tangentially to the scalp at an angle of 45° to the midsagittal plane with the handle pointing laterally and posteriorly. Electromyographic (EMG) activity was recorded from the right ADM with Ag–AgCl electrodes attached in a belly–tendon montage. Signals were filtered (30 Hz to 2 kHz), amplified (Digitimer 360; Digitimer Ltd., Hertfordshire, UK), stored on a computer via a Power 1401 data acquisition interface (Cambridge Electronic Design Limited, Cambridge, UK) and analyzed using Signal Software (Cambridge Electronic Design Limited, Cambridge, UK).

### 2.3. Nonfocal Plasticity Induction by tDCS (Experiment 1)

For tDCS, the current was delivered using a pair of 35 cm^2^ rubber electrodes covered with saline-soaked sponges connected to a battery-driven, constant-current stimulator (DC-STIMULATOR PLUS, NeuroConn Gmbh, Ilmenau, Germany). The target electrode was positioned over the right ADM M1 representation area and the reference electrode above the right supraorbital area. The injected current was 1mA and delivered continuously for 13 min for anodal tDCS and 9 min for cathodal tDCS, which induces cortical excitability alterations lasting for ~1 h after the end of stimulation [10,31]. The current was slowly ramped-up and down over 10 s at the beginning and end of the stimulation period, respectively. Electrode impedances were kept below 10 kΩ to minimize the tingling skin sensation. All stimulation parameters conformed to the safety guidelines for tDCS [32].

### 2.4. Focal Plasticity Induction by PAS (Experiment 2)

For PAS, single-pulse TMS of the left M1 was combined with peripheral nerve stimulation (Digitimer D185 stimulator; Digitimer) that delivered electric pulses to the right ulnar nerve at the wrist level (cathode proximal, square waveform of 0.2 ms duration). TMS stimulation intensity was set to the percentage of maximum stimulator output (%MSO) which generated an MEP amplitude of ~1 mV, while peripheral nerve stimulation intensity was set three times higher than the individual sensory-perceptual threshold. During stimulation, the electrical stimulus over the peripheral nerve was followed by the TMS stimulus at an interstimulus interval of 10 ms (inhibitory PAS: PAS10) for LTD-like plasticity induction or 25 ms (excitatory PAS: PAS25) for LTP-like plasticity induction. Ninety pairs of stimuli were administered at a frequency of 0.05 Hz for 30 min [4,5].

### 2.5. Pharmacological Intervention

For each experimental session, either low (0.1 mg), medium (0.2 mg), or high (0.3 mg) dosages of apomorphine or a placebo drug (saline solution) were injected subcutaneously on the participants’ abdomen ten minutes before the start of the plasticity-inducing protocols. At the time of plasticity induction, apomorphine has reached peak plasma concentrations and has prominent effects on brain functions [26]. The levodopa equivalent dose (LED) estimate for apomorphine dosages was determined using the online dose calculator for Parkinson’s Disease (https://www.parkinsonsmeasurement.org/toolBox/levodopaEquivalentDose.htm, accessed on 6 January 2021). The LED of low-dose, medium-dose and high-dose apomorphine is 1 mg, 2 mg and 3 mg, respectively. The maximum dosage was set at 0.3 mg because relevant systemic side effects such as nausea and vomiting at higher doses were observed during a pilot testing on four young, healthy volunteers. To prevent adverse effects, the participants took 20 mg of the peripheral-acting dopaminergic antagonist domperidone orally three times per day for two days before the experiment and 2 h before apomorphine injection. This dosage of domperidone alone exerts no effects on motor cortical excitability [33].

### 2.6. Experimental Procedures

**Figure 1:**
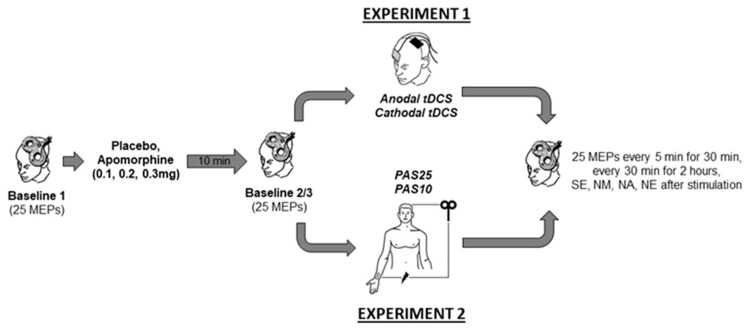
Time course of the experiments. Single-pulse TMS-evoked MEPs over the right ADM motor hotspot were recorded at intensities that evoked 1mV MEP amplitudes before drug injection (Baseline 1). Baseline 2 was recorded ten minutes after drug injection to look for drug-induced changes of cortical excitability. In case of any MEP alterations from baseline 1, Baseline 3 was recorded by adjusting the stimulator output to obtain a mean MEP amplitude of 1 mV. Then tDCS (anodal or cathodal) in Experiment 1 or PAS (PAS25 or PAS10) in Experiment 2 was administered, immediately followed by MEP after-measurements covering 120 min. Additional after-measurements were performed at the same evening (SE) and the morning at approximately 9:00 AM (NM), noon, at approximately 12:00 PM (NN), and evening, at approximately 6:00 PM (NE) of the second day after plasticity induction. Motor evoked potential (MEP). Transcranial direct current stimulation (tDCS). Paired associative stimulation (PAS).

Experiment 1 and 2 were conducted in a double-blinded, randomized, and placebo-controlled design. In all experimental sessions, another team member administered the drugs, and neither the experimenter nor the participants were aware of the dosage. In Experiment 1 with a mixed between- and within-subject design, each participant completed four randomized experimental sessions corresponding to the drug dosages combined with either anodal or cathodal tDCS. In Experiment 2, with a complete crossover design, each participant completed eight experimental sessions corresponding to the drug dosages combined with PAS10 and PAS25 stimulation. The experimental conditions were counterbalanced between subjects and were separated by an interval of at least one week to avoid interference effects. Except for the stimulation protocols (tDCS or PAS), both experiments’ procedures were identical (Figure 1). During the experiments, the participants were seated on a reclining chair with head and arm support and were asked to relax and keep their eyes open. EMG electrodes were placed at the right ADM using a belly-tendon montage. The locations of the EMG electrodes were marked to ensure their constant positioning throughout the experiment. Subsequently, the M1 representation (hotspot) of the ADM was determined by medium intensity TMS. The coil position where TMS with this given intensity elicited consistently the largest MEP amplitudes was defined as a hot spot and marked with a skin marker. Afterwards, the TMS intensity which induced an MEP amplitude of about 1 mV was identified. Using the same TMS intensity, 25 MEPs were recorded and considered as Baseline 1. Immediately after Baseline 1 was obtained, the participants received either apomorphine or the placebo substance. After 10 min, 25 MEPs were again recorded (Baseline 2) to control for possible drug-induced MEP amplitudes changes. If Baseline 2 differed relevantly (<0.2 or >0.2 mV) from Baseline 1, TMS intensity was readjusted to produce stable MEP amplitudes of ~1 mV (Baseline 3). Then anodal tDCS (13 min) or cathodal tDCS (9 min) was applied in Experiment 1 and PAS25 or PAS10 was applied for 30 min in Experiment 2. After stimulation, 25 MEPs were recorded every 5 min for half an hour and then every 30 min for 2 h. Additional measurements were conducted on the evening of the same day, and the morning, noon, and evening of the next day (Figure 1).

### 2.7. Data Analysis and Statistics

The MEPs were first visually inspected and discarded if preceded by significant EMG activities. The average MEP amplitude and %MSO were calculated for Baselines 1, 2, and 3 and average MEP amplitudes were calculated for all time points after plasticity induction for each individual and were analyzed via ANOVAs. First, to exclude pre-intervention between-session differences of corticospinal excitability, a one-way ANOVA was performed separately for Baseline 1 average MEPs and %MSO. The stimulation condition served as a between-subject factor (anodal tDCS, cathodal tDCS, PAS25, and PAS10). A similar ANOVA was performed on Baseline 3 average MEPs and %MSO. However, since this measurement was taken after drug administration, a within-subject factor drug condition (placebo, low-dose, medium-dose, and high-dose) was added to the model. Second, to explore the impact of apomorphine on pre-intervention within-session corticospinal excitability changes, Baseline 1 and 2 average MEPs were analyzed using an ANOVA with the within-subject factors time (Baseline 1 and 2) and drug condition (placebo, low-dose, medium-dose and high-dose). The participants’ %MSO was analyzed using the same type of ANOVA. However, for this ANOVA, the participants’ Baseline 1 and 3 %MSO was explored for the within-subject factors time and drug condition.

Concerning the post-intervention measurements, MEPs were normalized to Baseline 2 if Baseline 2 did not differ significantly from Baseline 1, and to Baseline 3 in participants where stimulation intensity had been adjusted. The normalized MEP amplitudes from all participants were pooled and averaged for each session and time point combination. The homogeneity of variance and sphericity were tested with Levene’s test (Experiment 1) and Mauchly’s test (Experiment 2), respectively. For Experiment 1, the MEPs were entered into a mixed model ANOVA with the between-subject factor stimulation (anodal and cathodal) and within-subject factors dosage (low, medium, high dose and placebo) and time (before stimulation, 0, 5, 10, 15, 20, 25, 30, 60, 90, and 120 min, same day evening, next morning, next afternoon, and next evening after stimulation). For Experiment 2, we conducted a repeated-measures ANOVA with the within-subject factors stimulation (PAS10 and PAS25), dosage and time. All significant results of the ANOVAs were explored using post hoc comparisons (Bonferroni-corrected, paired, two-tailed *t*-tests, *p* = 0.05). Except when stated otherwise, all presented data are expressed as the mean ± standard error of the mean (SEM).

## 3. Results

All participants tolerated the stimulation procedures well. However, some side effects of medication emerged. In Experiment 1, several participants experienced fatigue (low dose: 5, medium-dose: 8, high dose: 8), dry mouth and throat (low dose: 5, medium-dose: 1), increased salivation (high dose: 5), nausea (low dose: 5, medium-dose: 4, high dose: 5), and vomiting (medium dose: 1, high dose: 2). In Experiment 2, 5 participants experienced nausea, and 4 participants vomited after receiving medium and high dosages of the substance, respectively. All symptoms immediately alleviated, and no participants required medical interventions or requested to terminate their participation.

### 3.1. Effect of Apomorphine on Baseline Corticospinal Excitability

The results of the ANOVAs performed on Baseline 1 average MEPs and %MSO, as well as Baseline 3 average MEPs and %MSO (Table 1), revealed no significant results (Supplementary Table S1). These findings indicate that pre-intervention corticospinal excitability was comparable between sessions (Supplementary Figure S1). Similarly, the ANOVA performed on Baseline 1 and 2 average MEPs yielded no significant main and interaction effects (Supplementary Table S2). For the ANOVA performed on Baseline 1 and 3 %MSO, the main effect of time was significant (F (1, 47) = 5.460, *p* = 0.024, ƞₚ^2^ = 0.101) and may have been driven by the minor adjustment of stimulation intensity after drug administration. However, the non-significant time and drug condition interaction suggest that %MSO adjustment did not depend on drug dosage (Supplementary Table S2). Collectively, the results indicate that placebo and apomorphine (at the applied dosages) had no impact on corticospinal excitability before interventional brain stimulation.

### 3.2. Effect of Apomorphine on tDCS-Induced Neuroplasticity

**Figure 2:**
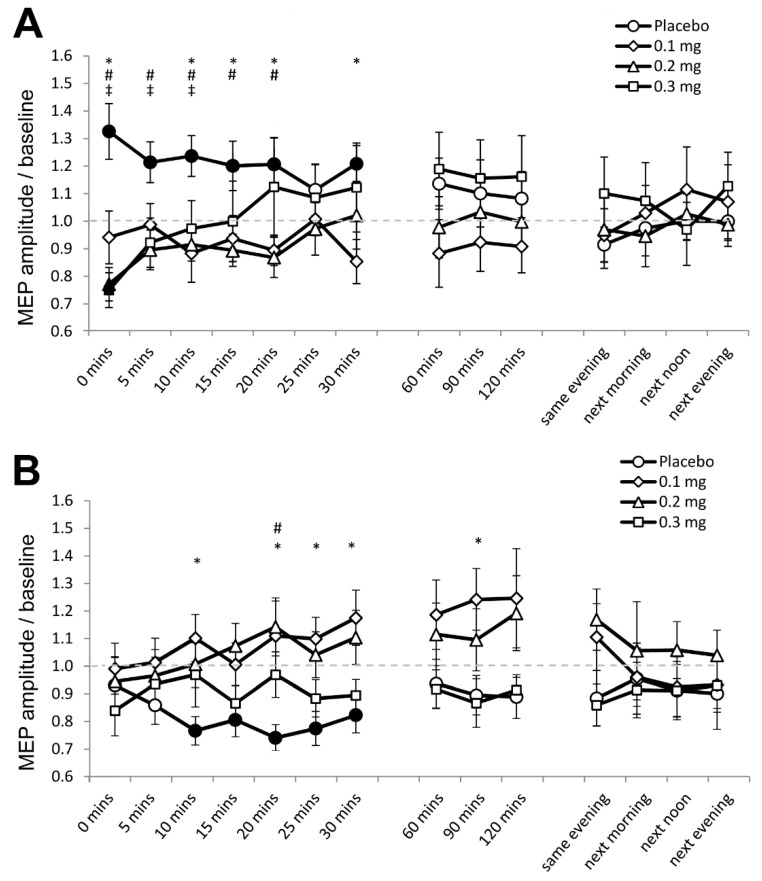
Effects of apomorphine on nonfocal plasticity induced by anodal and cathodal tDCS (Experiment 1). The *x*-axis displays the time points (in minutes) of post-stimulation measurements. The *y*-axis displays the mean MEP amplitudes after stimulation (MEPs were standardized to the corresponding baseline values for each participant). The graphs show that under placebo medication anodal tDCS enhanced excitability, and cathodal tDCS diminished excitability for ∼30 min after stimulation. (**A**) Low-dose (0.1 mg) abolished, medium-dose (0.2 mg) and high-dose (0.3 mg) transiently reversed the after-effect of anodal tDCS. (**B**) All dosages of apomorphine abolished the after-effect of cathodal tDCS. Filled symbols indicate statistically significant deviations of the post-tDCS MEP values compared with baseline. *, # and ‡ symbols indicate significant differences of the real medication compared with the placebo medication conditions at the same time points after plasticity induction (Bonferroni corrected, two-tailed, paired *t*-test, *p* ≤ 0.05). Error bars show standard error of mean (SEM). * 0.1 mg of apomorphine, # 0.2 mg of apomorphine, and ‡ 0.3 mg of apomorphine.

In Experiment 1, 0.81% (350) of the data set (43,200 MEPs) were excluded because significant spontaneous EMG activities preceded the respective MEPs. For the final data set, the assumption of homogeneity of variance (Levene’s test) was not violated (*p* ≥ 0.05). The ANOVA revealed a significant two-way interaction of stimulation and dose (F (3, 69) = 4.346, *p* = 0.007, ƞₚ^2^ = 0.165) and the three-way interaction of stimulation, dose and time was also significant (F (42, 966) = 2.053, *p* ≤ 0.001, ƞₚ^2^ = 0.085) (Table 2). Post hoc comparisons revealed that anodal tDCS increased cortical excitability under placebo medication, as indicated by the significantly larger MEP amplitude compared to baseline for 30 min after stimulation (Figure 2A). As compared to baseline, MEPs were moreover significantly smaller immediately after stimulation under medium-dose (*p* = 0.007) and high-dose apomorphine (*p* = 0.005). Furthermore, as compared to placebo medication, MEPs were significantly smaller for 30 min after stimulation under low dose, for the first 20 min after stimulation under medium-dose, and for the first 10 min after stimulation under high-dose apomorphine. These results indicate that the excitability enhancing effect of anodal tDCS was abolished under low-dose, and reversed to short-lasting excitability-diminishing plasticity under medium-dose and high-dose apomorphine.

For cathodal tDCS, the post hoc test results show that cortical excitability decreased as indicated by the significantly smaller MEPs relative to baseline for 30 min after stimulation under placebo medication (Figure 2B). In contrast, post-intervention MEPs in all apomorphine dosages did not significantly differ from respective baselines (Figure 2B). As compared to placebo medication, MEPs were significantly larger under the low-dose 10 min, 20 until 30 min, and 90 min after stimulation. For the medium-dose, MEPs were larger than those under placebo 20 min after stimulation, while for the high-dose, the MEPs were comparable to those under placebo (Figure 2B). Therefore, the excitability-diminishing effect of cathodal tDCS was abolished by all dosages of apomorphine.

### 3.3. Effect of Apomorphine on PAS-Induced Neuroplasticity

**Figure 3:**
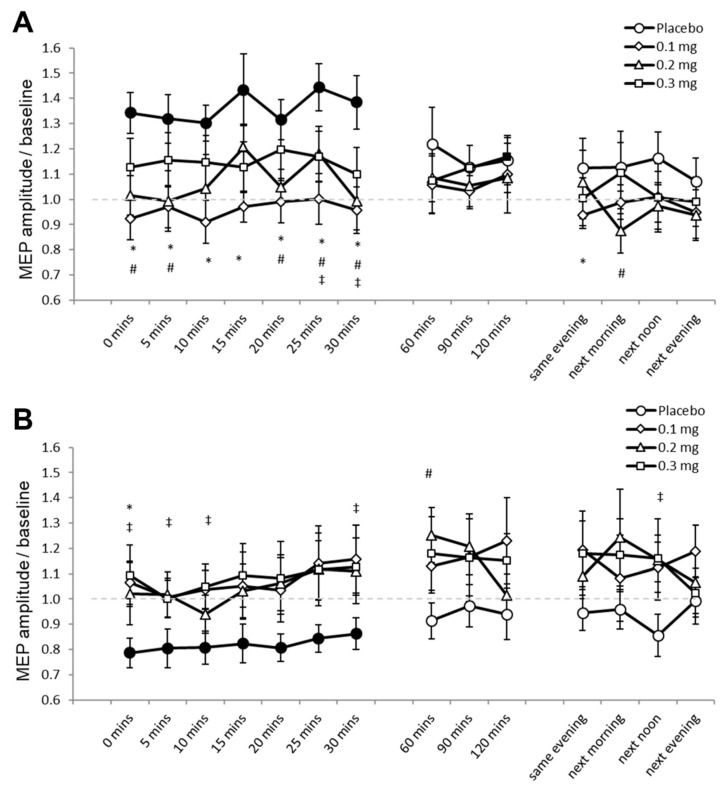
Effects of apomorphine on focal neuroplasticity induced by PAS25 and PAS10 (Experiment 2). The *x*-axis displays the time points (in minutes) of post-stimulation measurements. The *y*-axis shows the mean MEP amplitudes after stimulation (MEPs were standardized to the corresponding baseline values for each participant). The graphs show that, under placebo, excitatory (PAS25) and inhibitory PAS (PAS10) induce excitability enhancements and reductions lasting for ∼30 min after stimulation, respectively. (**A**) Low-dose (0.1 mg), medium-dose (0.2 mg), and high-dose (0.3 mg) apomorphine abolished, or diminished the after-effects of PAS25. (**B**) All dosages of apomorphine abolished the after-effect of PAS10. Filled symbols indicate statistically significant deviations of the post-PAS MEP values compared with baseline. *, # and ‡ symbols indicate significant differences of the real medication compared with the placebo medication conditions at the same time points after plasticity induction (Bonferroni corrected, two-tailed, paired *t*-test, *p* ≤ 0.05). Error bars show standard error of mean (SEM). * 0.1 mg of apomorphine, # 0.2 mg of apomorphine, and ‡ 0.3 mg of apomorphine.

For Experiment 2, 1.05% (453) of the data set (43,200 MEPs) were excluded because these were preceded by significant EMG activities. Mauchly’s test indicated that sphericity was not violated (*p* ≥ 0.05). The ANOVA revealed significant two-way interactions of stimulation and dose (F (3, 33) = 8.420, *p* ≤ 0.001, ƞₚ^2^ = 0.434), and stimulation and time (F (14, 154) = 2.319, *p* = 0.006, ƞₚ^2^ = 0.174) (Table 2). We performed the planned post hoc comparisons between drug dosages for all time points for both stimulation conditions to explore these interactions. Under placebo medication, PAS25 increased cortical excitability as indicated by the significantly larger MEP amplitudes compared to baseline for 30 min after stimulation (Figure 3A). MEPs did not significantly differ compared to respective baseline values under low-dose and medium-dose apomorphine, while MEPs were significantly larger than baseline 120 min (*p* = 0.049) after stimulation under high-dose apomorphine. Compared to the MEPs under placebo medication, MEPs under low-dose apomorphine were significantly smaller for 30 min and on the SE after stimulation. MEPs were significantly smaller than placebo for 30 min and on the NM and NA after stimulation under the medium-dose, while MEPs were significantly smaller than placebo 25 min and 30 min after stimulation under the high-dose. Therefore, the results indicate that the excitability-enhancing effect of PAS25 was abolished under low-dose and medium-dose, and diminished under high-dose apomorphine. PAS10 under placebo medication decreased cortical excitability as indicated by the significantly lower MEP amplitudes relative to baseline for 30 min after stimulation (Figure 3B). Post-intervention MEPs did not significantly differ to respective baselines in all apomorphine dosages. Compared to the MEPs under placebo medication, MEPs were significantly larger under low-dose (immediately after stimulation, *p* = 0.014), medium-dose (60 min after stimulation, *p* = 0.023), and high-dose (for 10 min and the 30th minute and NA after stimulation) apomorphine (Figure 3B). The results indicate that all dosages of apomorphine abolished the excitability-diminishing effect of PAS10.

## 4. Discussion

In the present study, we explored the impact of the nonselective dopamine agonist apomorphine on nonfocal and focal plasticity induced by tDCS and PAS, respectively. The results indicate that global dopamine receptor activation with apomorphine at the applied dosages has a deleterious impact on human M1 plasticity. Apomorphine either abolished, diminished, or reversed LTP-like plasticity. Furthermore, all dosages of apomorphine abolished LTD-like plasticity. Therefore, the overall results essentially resemble the effect of low dose L-DOPA despite the differences in pharmacokinetics between the substances [20,21].

### 4.1. Impact of Apomorphine on LTP-Like Plasticity

Apomorphine had a general abolishing effect on nonfocal and focal LTP-like plasticity, albeit at different degrees. Low-dose apomorphine abolished the facilitatory effect of anodal tDCS and PAS25. In contrast, medium-dose and high-dose apomorphine reversed the facilitatory effect of anodal tDCS immediately after stimulation, while medium-dose abolished, and high-dose diminished the facilitatory effect of PAS25. The overall detrimental effect of apomorphine on LTP-like plasticity resembles that observed under low-dose L-DOPA [20,21]. This is not surprising since the LEDs of the applied apomorphine dosages are comparable to low-dose L-DOPA application in previous studies [34]. Therefore, the predominant mechanism behind the effect of all apomorphine dosages could be a direct activation of presynaptic D2 autoreceptors located at the soma and dendrites of midbrain dopamine neurons in the VTA and substantia nigra pars compacta (SNc), as well as at their axon terminals in M1 [35,36,37]. Presynaptic autoreceptors on nerve terminals directly modulate dopamine neuron activity by inhibiting their excitability and activating G-protein coupled inwardly rectifying potassium channels (GIRKs), and indirectly by altering dopamine uptake, reduce the probability of dopamine synthesis and vesicular release [27,35,38,39,40,41]. GIRK channel activation at resting membrane potentials leads to potassium efflux that hyperpolarizes neurons and blocks cell firing [35,41]. Moreover, given that dopamine neurons can establish synapses that release glutamate [42], reducing freely available dopamine would result in decreased glutamatergic activity and calcium release needed for LTP induction [43]. Behaviorally, the administration of apomorphine at low doses causes locomotor hypoactivity and the suppression of exploration in rats, which is considered to be related to an impairment of, in particular, the mesolimbic/cortical dopamine transmission [44].

For the medium, and high dosages, nonfocal LTP-like plasticity was reversed into short-lasting inhibition. This medium- and high-dose apomorphine effect is qualitatively reminiscent of the reduced excitability under medium-dose L-DOPA that lasted for up to the next evening after anodal tDCS [12,20]. However, its duration is much shorter. The shorter duration of this effect compared to medium dosages L-DOPA can be explained by the fact that the respective apomorphine dosages are still more in the low dosage L-DOPA equivalent range, thus a mixed effect on D2 autoreceptors and postsynaptic D2 receptors might be assumed. Postsynaptic D2 receptor activation decreases surface α-amino-3-hydroxy-5-methyl-4-isoxazole propionic acid (AMPA) receptor level and attenuates AMPA currents [45,46]. D2 receptors further limit somatic excitability by decreasing L-type calcium currents [47,48,49]. Moreover, while D2 receptors do not directly modulate the depolarizing currents contributed by NMDA receptors, they limit the activation of calcium-dependent pathways due to a PKA-dependent decrease in calcium influx through NMDA receptors [49,50]. The overall reduction of intracellular calcium concentration will then favor the induction of LTD-like effects, because this kind of plasticity requires lower amounts of intraneuronal calcium than those required for LTP-like plasticity [43].

Medium-dose and high-dose apomorphine, on the other hand, abolished and diminished PAS-induced focal LTP-like plasticity, respectively, but in difference to tDCS, they did not reverse excitability-enhancing to excitability-diminishing effects. These effects differ from those of L-DOPA, which in previous studies facilitated PAS-induced LTP-like plasticity under medium dosage, and converted it to LTD-like plasticity under high dosage application [12,21]. The effect differences in relation to L-DOPA are likely caused by the fact that all apomorphine dosages applied in the present study were equivalent to the low dosage range of L-DOPA, as outlined in detail above. The gradually discernable effect of medium-dose and high-dose apomorphine on focal and nonfocal LTP-like plasticity is likely caused by the fact that, at the synaptic level, PAS25 is assumed to induce more robust effects (suprathreshold synchronous activation) than anodal tDCS (subthreshold membrane potential alteration) [51]. Thus, a minor reduction of NMDA receptor activity by D2 receptor activation would not be sufficient to reverse the LTP-like effects generated by PAS25.

### 4.2. Impact of Apomorphine on LTD-Like Plasticity

In contrast to LTP-like plasticity, all dosages of apomorphine abolished focal and nonfocal LTD-like plasticity. The abolishment of LTD-like plasticity under low-dose apomorphine resembles that observed under low-dose L-DOPA and bromocriptine [20,22,52]. For the medium-dose, the abolishment of nonfocal and focal LTD-like plasticity differs, however, from those obtained under medium-dose L-DOPA and medium-dose application of the D2 receptor agonist bromocriptine, which both enhanced LTD-like plasticity [20,22,52]. Concerning the high-dose, the direction of effect was in line with the effect of high-dose L-DOPA and high-dose D2 receptor agonist bromocriptine on nonfocal and focal LTD-like plasticity [20,22,52]. The relatively low dosage range of apomorphine compared to L-DOPA again explains why LTD-like plasticity was abolished regardless of the plasticity induction protocol under apomorphine. Animal studies showed that activation of D1 and D2, metabotropic AMPA glutamatergic and cholinergic receptors are required for LTD induction [53]. Indeed, dopamine-facilitated LTD depends on mGluR activation and synergistic activation of MAPK, as well as cooperative activation of D1 and D2 receptors [54,55,56]. As we argued already for the effect of dopaminergic enhancement on LTP-like plasticity, at the dosage range applied in the present study, the primary locus of action of apomorphine might be inhibitory presynaptic D2 dopamine receptors, causing diminished dopaminergic activity and extracellular dopamine levels [27,35]. In accordance, other studies conducted in healthy humans highlight the importance of postsynaptic dopaminergic receptor activation for LTD-like plasticity. D2 receptor blockade with sulpiride abolished the LTD-like after-effects of continuous TBS and cathodal tDCS [13,14].

### 4.3. General Remarks

The present results indicate that the non-selective dopamine receptor agonist apomorphine at the applied dosages is detrimental for plasticity induction in M1. This effect is different from those of previous dosage-titration studies with L-DOPA, which facilitates plasticity induction in the human M1 when given at optimal doses. Collectively, these findings further support the nonlinear dosage-dependent effect of dopamine receptor activation on plasticity. Although a comparison with respect to L-DOPA-equivalent medium and high-dosages was not possible due to tolerability problems, the impact of the applied dosages of apomorphine on plasticity likely represents one side of the inverted U-shape dose-response function of dopamine receptor activation: too low and too high activity impairs, while optimal dose facilitates plasticity. However, we cannot conclude from the results of the present study if the facilitatory effects of dopamine on plasticity, as obtained by L-DOPA, depend on phasic dopamine release, because we were not able to apply the respective LED of apomorphine.

The results of the present study, together with those obtained by global dopamine receptor activation with 100 mg L-DOPA [20,21], selective enhancement of D2 receptor activity with 10 mg bromocriptine (LED: 100 mg) [22], and D1-like receptor activation with 100 mg L-DOPA under the D2 receptor antagonist sulpiride [23], collectively support the significant role of pre- and postsynaptic dopamine receptor activation on plasticity induction in humans. Postsynaptic receptor-mediated facilitation of M1 plasticity under optimal dopamine level may account for learning improvements in healthy subjects and stroke patients [57,58,59,60]. In contrast, presynaptic receptor-mediated inhibition of cortical plasticity under low dosages may explain working and episodic memory performance impairments in healthy young human volunteers administered with dosages equivalent to those used in the present study [61,62]. In principle accordance with the plasticity-enhancing effects of optimal dosages of dopaminergic enhancement, L-DOPA facilitates M1 plasticity restoration [16,53,63], and working memory performance also in early stage PD patients [29]. Based on the results of the present study, and previous studies in healthy humans [20,21], this effect might critically depend on the applied dosage. Because of baseline differences of dopaminergic activity in healthy humans, and patients, the specific dosages which lead to improved plasticity might however differ, and thus require a direct exploration in patient studies.

Some limitations of the present study should be taken into account. First, the mechanistic explanation behind the results is speculative at present. However, in addition to evidence from animal studies, the results are supported by the findings from previous studies involving L-DOPA and selective dopamine agonists and antagonists [20,22,23,52]. Second, blinding might have been compromised in several participants for whom adverse effects such as nausea and vomiting were documented. However, in most participants, side effects, if present, occurred across different drug dosages; thus, blinding with respect to apomorphine dosages should have been maintained in these cases. Third, our study participants are different from those who participated in previous studies, particularly those involving L-DOPA [20,21]. This might limit the direct comparability of results because of individual differences in baseline dopamine. The demographic characteristics (e.g., all participants were within a specific relatively narrow age range) of the participants were reasonably comparable between studies. However, dopamine-related polymorphisms are associated with significant inter-individual differences in motor learning and the effect that dopaminergic drugs such as L-DOPA have on motor learning and M1 plasticity [64]. This issue, however, is not yet fully explored concerning dopamine agonists. Fourth, the sample sizes in Experiment 1 (24) and Experiment 2 (12) are relatively small. Although the number of participants was comparable with those of previous L-DOPA studies [20,21], and was not statistically underpowered for the primary statistical tests (the respective ANOVAs), considering the variability of dopamine effects, and to substantiate the results of the exploratory post-hoc tests further, replication studies in larger populations are warranted. Finally, in terms of LED, a direct comparison of the results of the present study with those from earlier studies with L-DOPA faces limitations, because the LEDs of apomorphine used in the present study were all in the low range of L-DOPA dosages applied in previous studies. Accordingly, the major effect of apomorphine in the present study, an abolishment of stimulation-induced plasticity, resembles what is seen under low dose L-DOPA. In the future, the impact of other agonists on plasticity should be explored, particularly those with respective LED of medium and high L-DOPA dosages, and less prominent adverse effects.

## 5. Conclusions

Our results demonstrate that apomorphine at low LED is detrimental to M1 plasticity, similar to low-dose L-DOPA. These findings support the nonlinear dosage-dependent effect of dopamine receptor activation on neuroplasticity in humans, in particular the inhibitory effect at low dosages. However, we cannot yet conclude whether apomorphine at dosages equivalent to the optimal L-DOPA dose will facilitate plasticity. A better understanding of how nonselective dopamine agonists affect plasticity and cognitive functions may in the future advance our knowledge of the pharmacotherapeutic effect of dopaminergic medications in neurological and psychiatric disorders.

## Figures and Tables

**Table 1:** Prestimulation peak-to-peak MEP amplitude and TMS intensity before and after application of apomorphine.

Medication Condition	Baseline 1		Baseline2		Baseline 3	
	MEP (mV)	MSO (%)	MEP (mV)	MSO (%)	MEP (mV)	MSO (%)
Anodal tDCS						
Placebo	0.97 ± 0.12	47.08 ± 7.61	0.94 ± 0.21	47.08 ± 7.61	0.96 ± 0.14	47.75 ± 7.52
0.1 mg	1.00 ± 0.11	48.92 ± 10.35	1.03 ± 0.28	48.92 ± 10.35	1.03 ± 0.11	49.00 ± 10.20
0.2 mg	0.99 ± 0.10	48.75 ± 8.95	0.87 ± 0.26	48.75 ± 8.95	1.01 ± 0.13	49.42 ± 9.12
0.3 mg	0.97 ± 0.10	49.58 ± 9.52	0.88 ± 0.54	49.58 ± 9.52	0.98 ± 0.09	50.42 ± 9.70
Cathodal tDCS						
Placebo	1.00 ± 0.07	49.17 ± 10.50	0.90 ± 0.07	49.17 ± 10.50	0.95 ± 0.10	49.17 ± 9.78
0.1 mg	1.00 ± 0.08	48.08 ± 8.06	0.82 ± 0.20	48.08 ± 8.06	0.96 ± 0.09	50.25 ± 8.74
0.2 mg	0.99 ± 0.11	49.50 ± 9.66	1.01 ± 0.34	49.50 ± 9.66	1.02 ± 0.12	50.17 ± 10.03
0.3 mg	1.02 ± 0.11	48.75 ± 9.55	1.02 ± 0.21	48.75 ± 9.55	1.01 ± 0.12	48.42 ± 9.98
PAS25						
Placebo	1.05 ± 0.13	48.83 ± 7.36	1.00 ± 0.15	48.83 ± 7.36	1.03 ± 0.13	49.33 ± 7.00
0.1 mg	1.06 ± 0.15	48.08 ± 7.86	1.13 ± 0.36	48.08 ± 7.86	1.03 ± 0.07	47.58 ± 8.25
0.2 mg	1.02 ± 0.08	47.67 ± 8.84	1.06 ± 0.23	47.67 ± 8.84	1.01 ± 0.09	48.42 ± 8.91
0.3 mg	1.05 ± 0.06	47.08 ± 8.02	1.03 ± 0.16	47.08 ± 8.02	1.08 ± 0.14	46.75 ± 8.47
PAS10						
Placebo	1.01 ± 0.14	47.50 ± 7.43	1.05 ± 0.34	47.50 ± 7.43	1.05 ± 0.34	47.50 ± 7.40
0.1 mg	1.06 ± 0.16	47.42 ± 6.47	1.02 ± 0.27	47.42 ± 6.47	1.02 ± 0.27	47.42 ± 6.47
0.2 mg	0.93 ± 0.13	49.00 ± 7.93	0.94 ± 0.24	49.00 ± 7.93	0.94 ± 0.24	49.42 ± 7.86
0.3 mg	1.00 ± 0.16	50.42 ± 8.59	1.01 ± 0.24	50.42 ± 8.59	1.06 ± 0.22	50.58 ± 8.32

Mean MEP amplitudes and stimulation intensities [percentage of maximum stimulator output (%MSO); mean + SD] of baseline 1–3.

**Table 2:** Results of the ANOVAs performed for Experiment 1 and 2.

	Numerator df	Denominator df	*F*-Value	*p*-Value	ƞᵨ^2^
**Experiment 1 (tDCS)**					
Stimulation	1	22	0.763	0.392	0.034
Dose	3	69	0.153	0.927	0.007
Time	14	322	0.954	0.500	0.042
Stimulation × dose	3	69	4.346	0.007 *	0.165
Stimulation × time	14	323	0.384	0.979	0.017
Dose × time	42	966	0.935	0.590	0.041
Stimulation × dose × time	42	966	2.053	<0.001 *	0.085
**Experiment 2 (PAS)**					
Stimulation	1	11	0.576	0.464	0.050
Dose	3	33	0.228	0.876	00.020
Time	14	154	1.405	0.157	0.113
Stimulation × dose	3	33	8.420	<0.001 *	0.434
Stimulation × time	14	154	2.319	0.006 *	00.174
Dose × time	42	462	0.438	0.999	0.038
Stimulation × dose × time	42	462	0.993	0.487	0.083

The ANOVA encompasses the time course of the MEP measures up to the next evening after stimulation. * = indicate significant results (*p* < 0.05), df = Degrees of freedom, tDCS = transcranial direct current stimulation, PAS = paired associative stimulation.

## Data Availability

The data presented in this study are available on request from the corresponding author. The data are not publicly available due to ethical restriction.

## Supplementary Materials

The following are available online at https://www.mdpi.com/article/10.3390/pharmaceutics13050718/s1, Table S1: Results of the ANOVAs performed for Baseline 1 and Baseline 3 on average MEPs and %MSO. Table S2: Results of the ANOVAs performed on Baseline 1 and 2 average MEPS, and Baseline 1 and 3 %MSO. Figure S1: Prestimulation peak-to-peak MEP amplitude before and after application of apomorphine.
