# Supplementary material for: Inhibitory Effect of Apomorphine on Focal and Nonfocal Plasticity in the Human Motor Cortex
Source: Pharmaceutics. 2021 May 13;13(5):718. doi: 10.3390/pharmaceutics13050718 (PMC8153161; doi:10.3390/pharmaceutics13050718)
Supplement: Supplementary file 1 [file pharmaceutics-13-00718-s001.zip › pharmaceutics-1198399-supplementary.pdf]

pharmaceutics

Supplementary Materials: Inhibitory Effect of Apomorphine on Focal and Nonfocal Plasticity in the Human Motor Cortex

Shane M. Fresnoza, Giorgi Batsikadze, Lynn Elena Müller, Constanze Rost, Michael Chamoun, Walter Paulus, Min-Fang Kuo and Michael A. Nitsche

Table S1. Results of the ANOVAs performed for Baseline 1 and Baseline 3 on average MEPs and %MSO.

	df	*F*-value	*p*-value
**Baseline 1 MEPs**			
Stimulation	2	1.389	0.252
**Baseline 1 %MSO**			
Stimulation	2	0.093	0.911
**Baseline 3 MEPs**			
Dosage	3	1.036	0.382
Stimulation	2	0.746	0.480
Dosage × stimulation	6	0.768	0.597
**Baseline 3 %MSO**			
Dosage	3	0.806	0.493
Stimulation	2	0.087	0.917
Dosage × stimulation	6	1.145	0.340

df = Degrees of freedom, MEP = motor evoked potential, MSO = maximum stimulator output.

Table S2. Results of the ANOVAs performed on Baseline 1 and 2 average MEPS, and Baseline 1 and 3 %MSO.

	Numerator df	Denominator df	*F*-value	*p*-value	η_p^2
**MEPs**					
Time	1	47	1.303	0.259	0.027
Drug condition	3	141	0.750	0.542	0.016
Time × drug condition	3	141	0.060	0.981	0.001
**%MSO**					
Time	1	47	5.460	0.024*	0.104
Drug condition	3	141	0.877	0.455	0.018
Time × drug condition	3	141	0.983	0.403	0.020

* = indicates significant results ($p < 0.05$), df = Degrees of freedom, MEP = motor evoked potential, MSO = maximum stimulator output.

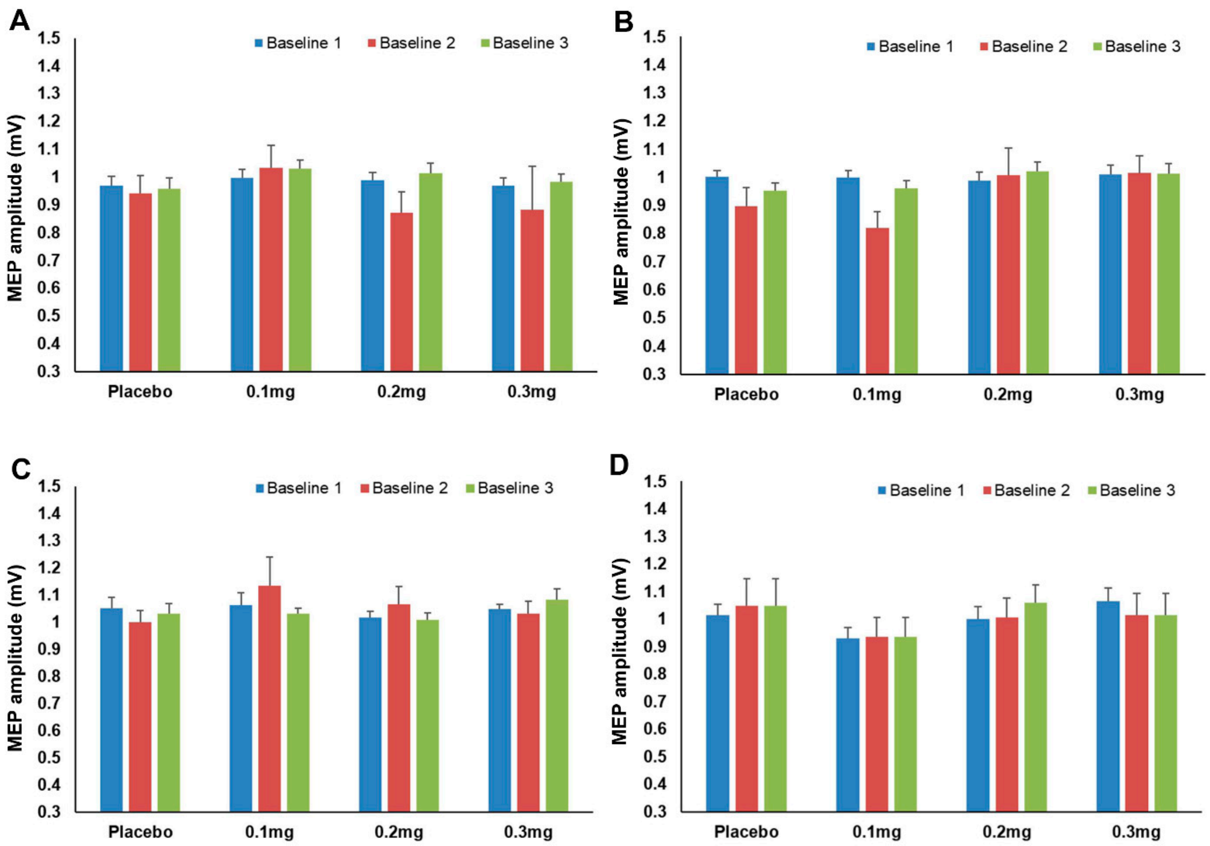

Figure S1. Prestimulation peak-to-peak MEP amplitude before and after application of apomorphine. Baseline 1 (before application of apomorphine), 2 and 3 (both after application of apomorphine) average MEPs before anodal tDCS **(A)**, cathodal tDCS **(B)**, PAS25 **(C)**, and PAS10 **(D)** stimulation. The x-axis displays the drug conditions. The y-axis displays the mean MEP amplitudes. There were no significant differences in the MEP amplitudes between drug conditions and between baseline measurements in all stimulation conditions.
